# Dietary Lycopene Intake and Gastric Cancer Risk: Findings from a Case-Control Study

**DOI:** 10.3390/nu18071143

**Published:** 2026-04-02

**Authors:** Ngoan Tran Le, Yen Thi-Hai Pham, Linh Thuy Le, Phuong M. Nguyen, Ninh Thi Nguyen, Minh Hoang Nhat Phuong, Chi Thuy Nguyen, Phong Gia Dang, Thao Thu Thi Vu, Nam S. Vo, Lang Wu, Tin C. Nguyen, Jennifer Cullen, Hung N. Luu

**Affiliations:** 1 Institute of Research and Development, Duy Tan University, Da Nang 550000, Vietnam; 2 Department of Clinical Research, Vinmec Healthcare System, Hanoi 100000, Vietnam; 3 UPMC Hillman Cancer Center, University of Pittsburgh Medical Center, Pittsburgh, PA 15232, USA; 4 Dr. Mary and Ron Neal Cancer Center, Houston Methodist Research Institute, Houston, TX 77030, USA; jcullen@houstonmethodist.org; 5 Laboratory of Embryology and Genetics of Human Malformation, Imagine Institute, INSERM UMR (National Institute of Health and Medical Research, Mixed Research Unit), 75015 Paris, France; lelinh2611@gmail.com; 6 Department of Computer Science and Software Engineering, Auburn University, Auburn, AL 36849, USA; pzn0021@auburn.edu; 7 Department of Occupational Health, Institute for Preventive Medicine and Public Health, Hanoi Medical University, Hanoi 100000, Vietnam; ninhnguyenydd@gmail.com (N.T.N.); hoangming2101@gmail.com (M.H.N.P.); nguyenthuychiwork@gmail.com (C.T.N.); giaphong.work@gmail.com (P.G.D.); 8 General Daycare Inpatient Department, Hanoi Oncology Hospital, Hanoi 100000, Vietnam; thaovudr198@gmail.com; 9 VinUni Big Data Research Institute, VinUniversity, Hanoi 100000, Vietnam; nam.vs@vinuni.edu.vn; 10 Department of Interdisciplinary Oncology, LSU-LCMC Health Cancer Center, Louisiana State University Health Sciences Center, New Orleans, LA 70112, USA; lwu3@lsuhsc.edu; 11 Department of Computer Science, Wayne State University, Detroit, MI 48202, USA; tin@wayne.edu; 12 Karmanos Cancer Institute, Wayne State University, Detroit, MI 48201, USA; 13 Department of Population and Quantitative Health Sciences, Case Western Reserve University School of Medicine, Cleveland, OH 44106, USA; 14 Department of Medicine, Weill Cornell Medicine, Cornell University, New York, NY 10065, USA

**Keywords:** dietary lycopene, risk, gastric cancer, case-control study, Vietnam

## Abstract

**Background/Objectives**: Lycopene, a red lipophilic carotenoid hydrocarbon pigment found primarily in tomatoes and other red/pink fruits and vegetables, has anti-inflammatory, anticarcinogenic and cardioprotective properties. There is a lack of evidence regarding the effect of lycopene intake on gastric cancer risk in the Asian population. We, thus, evaluate the association between lycopene intake and gastric cancer risk in a hospital-based case–control study, including 1182 incident cases of gastric cancer and 2995 controls in Vietnam. **Methods**: Lycopene intake was derived from a semi-quantitative, validated food frequency questionnaire. An unconditional logistic regression model was performed to estimate the odds ratios (ORs) and 95% confidence intervals (CIs) for gastric cancer risk in relation to lycopene intake, adjusted for potential confounding factors. **Results**: Overall, there was a dose–response inverse association between lycopene intake and gastric cancer risk (OR_per-SD increment_ = 0.88, 95% CI: 0.81–0.95; *P_trend_* = 0.002). Compared with quintile 1 (the lowest quintile), the ORs and 95% CIs of gastric cancer for quintiles 2, 3, 4 and 5 of the lycopene intakes were 0.63 (0.51–0.79), 0.64 (0.51–0.80), 0.65 (0.52–0.81) and 0.62 (0.50–0.78), respectively. A similar pattern of inverse association between lycopene intake and gastric cancer risk was seen only in females, ever alcohol drinkers, and individuals with *H. pylori* negative status, without type 2 diabetes and with blood group B (all *P_heterogeneity_* > 0.05). **Conclusions**: We observed a reduced risk of gastric cancer in individuals with higher lycopene intake in the Vietnamese population, regardless of BMI or smoking status. Our results have great implications for a healthy dietary pattern (i.e., lycopene with major sources from fruits and vegetables) for strategies in the prevention and control programs of gastric cancer in low-and middle-income countries.

## 1. Introduction

Globally, gastric cancer is one of the leading malignancies, both in incidence and mortality. Recent data from the 2022 GLOBALCAN showed that gastric cancer is ranked fifth in incidence and mortality, with approximately 1 million new cases and 660,999 related deaths in 2022 [1]. In 2025, there were 30,300 new cases of gastric cancer and 17,720 related deaths in the United States [2]. Gastric cancer is also one of the leading cancers in Vietnam, where it is ranked fourth in incidence and third in mortality (16,277 new cases and 13,264 deaths in 2022) [3]. Established risk factors for gastric cancer include those of non-modifiable factors, including age, sex, and genetics, and those of modifiable factors, including cigarette smoking, alcohol drinking, *H. pylori* infection, and diet [4,5].

Lycopene is a red lipophilic carotenoid hydrocarbon pigment with main sources from tomatoes and other red or pink fruits and vegetables, including orange, grapes, carrots, cranberries or apricots [6]. Being one of the most effective free radical scavengers in vitro of all carotenoids, lycopene increases the levels of glutathione and activities of antioxidant enzymes that help protect DNA, lipids and other macromolecules [7,8]. Besides its antioxidant properties, experimental studies also showed that lycopene has other properties, such as anti-inflammatory [9], anticarcinogenic and cardioprotective [10], suggesting its potential beneficial roles in chronic diseases, including cancer, cardiovascular disease and metabolic syndrome.

Using data from the Singapore Chinese Health Study, an ongoing prospective cohort study of more than 63,000 Chinese Singaporeans, we recently showed that tomato intake was associated with reduced risk of hepatocellular carcinoma (hazard ratio-HR = 0.98, 95% confidence interval-CI: 0.97–0.99; *P_trend_* = 0.0004) but not lycopene intake (*P_trend_* = 0.54) [11]. Observational data from a recent umbrella review reported that there was an inverse association between dietary lycopene intake or serum lycopene with risks of prostate cancer, stroke, metabolic syndrome, cardiovascular disease, male infertility and all-cause mortality [12]. Also, in a meta-analysis published in 2013, comprising four case–control studies (i.e., 839 gastric cancer cases versus 1309 controls) and three cohort studies (i.e., 641 incident cases of gastric cancer versus 112,235 non-cases), Yang et al. [13] showed that there was a null association between lycopene intake and risk of gastric cancer (pooled relative risk-RR = 0.88, 95% confidence interval-CI: 0.67–1.16), both in the case–control study design (pooled OR = 0.87, 95% CI: 0.52–1.45) and in the cohort study design (pooled OR = 0.87, 95% CI: 0.68–1.12). Notably, these studies were conducted in the U.S. [14], Europe [15,16,17,18,19], and Uruguay in Latin America [20], but not in Asian countries; there was a significant heterogeneity among included studies in this meta-analysis (Q = 14.81, *p* = 0.022, *I*^2^ = 59.48%) [13]. The incidence of gastric cancer in North America (5.1/100,000 population in males-3.6/100,000 population in females) and Europe (7.7/100,000 population in males-3.6/100,000 population in females) is much lower than that in Asia (23.0/100,000 population in males-9.7/100,000 population in females in Eastern Asia) [1]. It remains unclear the role of lycopene in gastric cancer risk among the Asian population.

We, therefore, determined the association between dietary lycopene intake and risk of gastric cancer in the Vietnamese population, using data from a hospital-based case–control study in Hanoi, Vietnam; a study comprising 1182 gastric cancer cases and 2995 controls.

## 2. Materials and Methods

### 2.1. Study Population

For the current analysis, we used data obtained from the hospital-based case–control study that was conducted in Hanoi, Vietnam. Its study design, methods, and initial results have been described in detail elsewhere [21,22,23]. Briefly, participants in the current study were recruited from four tertiary hospitals in Hanoi, Vietnam (i.e., Bach Mai Hospital, Viet Duc University Hospital, National Cancer Hospital, and Hanoi Medical University Hospital) between 2003 and 2019. The study participants were recruited in four sub-periods: (1) 2003–2006 period (n = 625 study participants); (2) 2006–2007 period (n = 1342 study participants); (3) 2008 period (n = 407 study participants); and (4) 2018–2019 period (n = 4902 study participants). All study participants agreed to provide written informed consent before participating in the study. The current study was approved by the Institutional Review Boards (IRBs) of Hanoi Medical University (#3918/HMUIRB) and the International University of Health and Welfare, Japan (#19-Ig-17).

#### 2.1.1. Recruitment of Gastric Cancer Cases

The recruitment of patients with gastric cancer has been described in detail elsewhere [24,25,26]. Briefly, patients with gastric cancer were recruited within a week prior to the surgery. Potential participants were identified by reviewing the list of patients with cancer who were scheduled for surgery and who met the inclusion criteria. The inclusion criteria included: (1) pathologically confirmed as gastric cancer; (2) physically able to undergo surgery; (3) agreed to participate in the study; and (4) able to provide information on the exposure data questionnaire. The exclusion criteria in our study were (1) individuals who did not agree to participate in the study; (2) individuals who changed their diet during the illness; and (3) individuals who could not provide exposure data in the questionnaire.

#### 2.1.2. Recruitment of Controls

We identified potential controls from the list of patients who were scheduled for different surgeries at the same hospitals where the patients with gastric cancer were recruited. The inclusion criteria for control recruitment included (1) individuals who agreed to participate in the study; (2) individuals who were able to provide exposure and related information; and (3) individuals who were not diagnosed with malignancy or any history of cancer. The exclusion criteria included (1) individuals who refused to participate in the study and (2) individuals who changed their diet due to the course of illness during the study [24,25,26].

We recruited more controls (n = 2995) than gastric cancer cases (n = 1182) to improve the power of the study from the same four hospitals [24,25,26].

### 2.2. Exposure Information Collection

Trained interviewers used a structured questionnaire to collect exposure information from the study participants before the surgery. The structured questionnaire included the following sections: (1) sociodemographic factors; (2) lifetime tobacco and alcohol use; (3) body height and weight; (4) dietary habits (see Dietary Assessment for details); (5) medical history; (6) family history of cancer; and (7) occupational exposures. Medical information was extracted from the medical records by the trained medical extractors, including the infections of hepatitis B, hepatitis C, or HIV, and/or *H. pylori* (if any).

### 2.3. Dietary Assessment

A validated semi-quantitative food frequency questionnaire (FFQ) was used to collect dietary information from the study participants. Our FFQ comprised 85 food items commonly consumed by the Vietnamese and accounted for more than 90% essential nutrients. We asked the study participants how frequently they consumed the food and food groups during the past 12 months. Six groups of frequent answers were listed, including “6–11 times/year”, “1–3 times/month”, “1–2 times/week”, “3–4 times/week”, “5–6 times/week”, and “1–3 times/day”. Subsequently, we asked the study participants the amount of food consumed, which was later grouped into three portions: (a) small, (b) medium, and (c) large. We used the Vietnamese Food Composition Database to calculate the daily intake, on average, of 95 nutrients and non-nutrient compounds, including lycopene [27].

The validity of the FFQ used in our study was validated in a validation study among 1327 individuals, which was conducted between October 2017 and December 2017. We used two 24 h dietary recalls (24-HDRs), once every weekday and once every three consecutive other days in the validation study. The correlation coefficients (*R*^2^) between the FFQ and 24-HDR were between 0.38 (protein) and 0.53 (energy) [28]. We also conducted a reproducibility study among 150 healthy participants using two FFQs, two to three weeks apart. The test–retest correlation coefficient (*R*^2^) for dietary lycopene was 0.39.

### 2.4. Assessment of Other Covariates

We used the same structured questionnaire to collect information on other covariates used in the current study, including body mass index (BMI), which was calculated as weight in kilograms divided by height in meters squared. BMI was then re-grouped into: <18.5, 18.5–22.9, 23–24.9, and ≥25 kg/m^2^. Individuals whose BMI was ≥ 23 kg/m^2^ were defined as overweight or obese, following the recommendation for the Asian population by the World Health Organization (WHO) [29,30]. Age was initially collected as a continuous variable and then re-grouped into 15–39, 40–49, 50–59, 60–69, and ≥70 years. The highest education level was re-grouped as primary, secondary, and high school or higher. Smoking status was categorized into never smokers and ever smokers, while alcohol drinking was grouped into never drinkers and ever drinkers (consisting of current and past drinkers). Similarly, coffee drinking status was grouped as never drinkers and ever drinkers (consisting of current and past drinkers). The history of type 2 diabetes was collected as yes and no groups. Blood type and *H. pylori* status information was extracted from the medical records.

### 2.5. Statistical Analysis

We calculated means and standard deviations (SDs) for continuous variables and counts and proportions for categorical variables. In descriptive analysis to describe the study participants’ characteristics, we conducted *t*-test (or ANOVA) for continuous variables and *χ*^2^ test for categorical variables to show the differences in the distribution of participants’ characteristics across five quintiles of lycopene and between cases and controls. We performed unconditional logistic regression models to evaluate the association between lycopene intake and risk of gastric cancer, generating odds ratios (ORs) and respective 95% CIs. Potential confounders that were included in the multivariable regression models were (1) age (15–39, 40–49, 50–59, 60–69 and ≥70), (2) sex (male vs. female), (3) education level (primary school/secondary school/high school or higher), (4) BMI (<18.5, 18.5–22.9, ≥23 kg/m^2^), (5) smoking status (never vs. ever smokers, consisting of current and past smokers), (6) alcohol consumption (never vs. ever drinkera), (7) coffee drinking, (8) history of diabetes (yes vs. no), (9) family history of cancer (yes vs. no), (10) enrollment periods (2003–2006, 2006–2007, 2008, and 2018–2019), (11) total energy intake (kcal/day), (12) *H. pylori* status, and (13) blood groups (A, AB, B, and O).

Stratified analyses were further performed by (1) sex (i.e., male vs. female), (2) histologic type (i.e., non-cardia vs. cardia), (3) BMI (<23 kg/m^2^ versus ≥ 23 mg/m^2^), (4) smoking status (i.e., never vs. ever smoker), (5) alcohol drinking (i.e., never vs. ever drinker), (6) history of type 2 diabetes (i.e., yes vs. no), (7) *H. pylori* infection status and (8) blood group. We used ordinal values of the quintiles of lycopene intakes to test for a linear trend for the association between lycopene intake and risk of gastric cancer. We also tested for interaction between sex, BMI, histologic type, smoking status, alcohol drinking status, history of type 2 diabetes, *H. pylori* infection status and blood group with lycopene intake in the association between lycopene intake and gastric cancer risk by adding the product terms of lycopene intake and such variables in the multivariable logistic regression models.

Stata statistical package (version 14.0; Stata Corp., College Station, TX, USA) was used to conduct all statistical analyses. Restricted cubical splines between lycopene intake and gastric cancer risk were generated using the *R* package (Version 4.5.2). *p* < 0.05 was used as a statistically significant threshold, and a two-sided test was used for all analyses.

## 3. Results

Compared with individuals in the 1st quintile of lycopene intake (the lowest quintile), individuals in the 5th quintile (the highest quintile) were younger, more likely to be male, had higher education level, had higher frequency of refrigerator owned, yet had higher BMI and higher frequency of ever smoking, as well as coffee drinking, higher frequency of history of type 2 diabetes, and higher energy intake (All *p*’s *<* 0.05). There was no difference in terms of distribution of family history of cancer, alcohol drinking status, blood groups and *H. pylori* infection status across quintiles of lycopene intake (Table 1).

Compared with control subjects, patients with gastric cancer were older, had a lower intake of lycopene, were more likely to be female, were less likely to own refrigerators, had a lower education level, were more likely to have a BMI < 18.5 kg/m^2^, and were more likely to have a family history of cancer. They were also more likely to be ever smokers and ever drinkers; however, they were less likely to be coffee drinkers. They had a lower frequency of history of type 2 diabetes, a lower level of total energy intake and were more likely to be with blood groups A and AB (all *p*’s < 0.05). No statistically significant difference was seen regarding the distribution of *H. pylori* infection status between cases and controls (*p* = 0.88) (Table 2).

**Figure 1:**
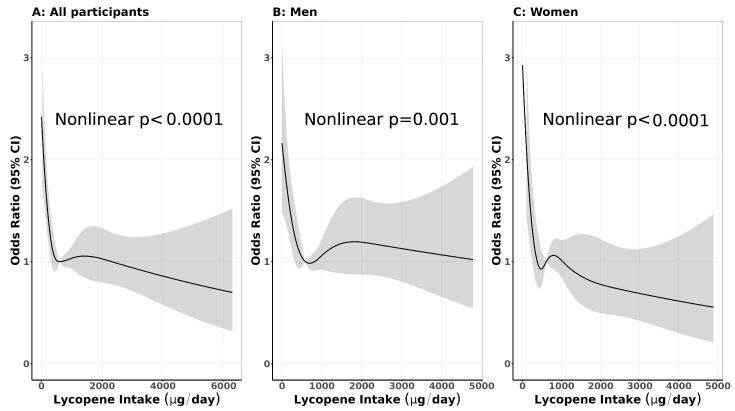
Restricted Cubical Splines on the Association Between Lycopene Intake and Gastric Cancer Risk, Overall and Stratified by Sex. (**A**) All participants; (**B**) men; and (**C**) women. Gray color is 95% confidence intervals of the odds ratios.

Overall, there was a dose–response inverse association between lycopene intake and gastric cancer risk (OR_per-SD increment_ = 0.88, 95% CI: 0.81–0.95; *P_trend_* = 0.002). Compared with quintile 1 (the lowest quintile), the ORs and respective 95% CIs of gastric cancer for quintiles 2, 3, 4 and 5 of the lycopene intakes were 0.63 (0.51–0.79), 0.64 (0.51–0.80), 0.65 (0.52–0.81) and 0.62 (0.50–0.78), respectively. A similar pattern was found among women (OR_per-SD increment_ = 0.80, 95% CI: 0.69–0.92; *P_trend_* < 0.001), but not among men (OR = 0.93, 95% CI: 0.84–1.02; *P_trend_* = 0.14; *P_heterogeneity_* = 0.13) (Table 3 and Figure 1).

In stratified analysis, the risk reduction pattern (between lycopene intake and gastric cancer risk) was seen in patients with non-cardia histologic type (OR_per-SD increment_ = 0.89, 95% CI: 0.82–0.96; *P_trend_* = 0.004); both individuals with a BMI < 23 kg/m^2^ (OR_per-SD increment_ = 0.91, 95% CI: 0.83–0.99; *P_trend_* = 0.03) and BMI ≥ 23 kg/m^2^ (OR_per-SD increment_ = 0.78, 95% CI: 0.63–0.96; *P_trend_* = 0.02) (*P_heterogeneity_* = 0.45); never smokers (OR_per-SD increment_ = 0.85, 95% CI: 0.77–0.95; *P_trend_* = 0.004), but not among ever smokers (OR_per-SD increment_ = 0.91, 95% CI: 0.80–1.02; *P_trend_* = 0.11) (*P_heterogeneity_* = 0.42); ever alcohol drinkers only (OR_per-SD increment_ = 0.83, 95% CI: 0.73–0.94; *P_trend_* = 0.003) (*P_heterogeneity_* = 0.05); individuals with *H. pylori* negative test results (OR_per-SD increment_ = 0.77, 95% CI: 0.62–0.95; *P_trend_* = 0.02) (*P_heterogeneity_* = 0.98); those without history of type 2 diabetes (OR_per-SD increment_ = 0.88, 95% CI: 0.81–0.97; *P_trend_* = 0.006), and individuals with blood group B (OR_per-SD increment_ = 0.81, 95% CI: 0.68–0.97; *P_trend_* = 0.02) (All *P’s_heterogeneity_* > 0.05) (Table 4). Because there were only 23 patients with gastric cancer with a history of type 2 diabetes, we could not conduct the stratified analysis in this sub-population.

## 4. Discussion

In the current analysis of a case–control study, comprising 1182 patients with gastric cancer and 2995 controls in Vietnam, we found a dose–response inverse association between lycopene intake and risk of gastric cancer. This reduction risk pattern was identified regardless of BMI or smoking status. However, the stratified analysis also revealed that this inverse association pattern was more apparent in females, ever alcohol drinkers, individuals with *H. pylori* negative status, those without a history of type 2 diabetes, and individuals with blood group B.

To our knowledge, the current study might be the first attempt at evaluating the association between dietary lycopene intake and gastric cancer risk in the Vietnamese population. Our main finding on the inverse association between lycopene intake and risk of gastric cancer is consistent with findings from a similar study design of a hospital-based case–control study in Uruguay, consisting of 120 gastric cancer cases and 360 controls (OR_tertile3vstertile1_ = 0.37, 95% CI: 0.19–0.73, *P_trend_* = 0.006) [20]. An interesting point here is that the incidence of gastric cancer in Uruguay (9.2/100,000 population) is higher than that in North America, Europe or some parts of South American countries, yet still lower than that in Vietnam (13.4/100,000 population) [1]. Our results, however, were not consistent with the meta-analysis conducted by Yang et al. [13], showing a null association between lycopene intake and gastric cancer risk. This meta-analysis included a case–control study in the U.S. (91 cases vs. 132 controls, OR_per SD increment_ = 0.70, 95% CI: 0.40–1.20) [14], a case–control study in France (354 cases vs. 354 controls, OR_Q4vs.Q1_ = 1.55, 95% CI: 0.91–2.64) [15], a case–control study in Poland (274 cases vs. 463 controls; OR_Q4vs.Q1_ = 1.19, 95% CI: 0.77–1.82) [16], a cohort study in the Netherland (282 incident cases vs. 3123 non-cases; HR = 1.00, 95% CI: 0.70–1.50) [17], a cohort study in Finland (220 incident cases vs. 27,110 non-cases; HR_Q4vs.Q1_ = 0.97, 95% CI: 0.41–2.34) [18], and a cohort study in Sweden (139 incident cases vs. 82,002 non-cases; HR_Q4vs.Q1_ = 0.92, 95% CI: 0.53–1.58) [19]. One of the major limitations in the meta-analysis by Yang et al. [13] was that there was a significant variation/heterogeneity in the above studies that were included in this analysis (Q = 14.81, *p* = 0.022, *I*^2^ = 59.48%).

The major sources of lycopene are tomatoes and other red or pink fruits and vegetables [6]. The same meta-analysis, conducted by Yang et al. [13] and included six case–control studies (1980 cases versus 2789 controls) and one cohort study (616 incident cases versus 4035 non-cases), all from either the U.S. or Europe, but one from Jiangsu, a province with a low prevalence of gastric cancer in China [32], showed that dietary tomato intake provided protective effect against the development of gastric cancer (pooled OR = 0.73, 95% CI: 0.60–0.90). Similarly, a recent meta-analysis, comprising 18 prospective cohort studies with a total sample size of 1,527,995 participants, aged between 18 and 90 years, reported that the consumption of fruits alone and in combination with vegetables was associated with a reduction in risk of gastric cancer by 13% (pooled RR = 0.87, 95% CI: 0.80–0.94) and 25% (pooled RR = 0.75, 95% CI: 0.61–0.93), respectively [33]. We also performed additional analysis by adjusting for fruits, vegetables or both in the multivariable logistic regression models and found that the pattern has not materially changed (Supplementary Table S1).

Extensive experimental studies showed that lycopene is involved in the modulation of multiple targets that are linked to carcinogenesis, including growth factors, transcriptional factors, cell survival, inflammatory pathways, angiogenesis and invasion (reviewed by Friedman M. [10]). For instance, lycopene performed as an in vivo redox agent, protecting tissues against the high concentration of reactive oxygen species (ROS), which leads to the inhibition of oxidative damage of cells and DNA [34]. Another study by Luo et al. [35] reported that up-regulation of antioxidants and immunity function in N-methyl-N′-nitro-N-nitrosoguanidine by lycopene treatment would have an anticancer effect against the development of gastric cancer in rats. Lycopene was also found to prevent smoke exposure-induced p53, p53 phosphorylation, p53 target genes, cell proliferation, and apoptosis in the gastric mucosa of ferrets via p21, Bax-1, caspase 3 and cyclin D1 [36].

Even though we found a reduction in gastric cancer risk among females, ever alcohol drinkers, individuals with *H. pylori* negative status, those without a history of type 2 diabetes and those with blood group B in stratified analysis, it appears that the differential difference only occurred by alcohol drinking status. Indeed, the ORs and 95% CIs for the never drinker group and the ever drinker group were 0.92 (0.63–1.02) and 0.83 (0.73–0.94), and the *P_heterogeneity_* = 0.05 or reached a statistically significant level. While this finding has a great implication for gastric cancer prevention and control programs, particularly among alcohol drinkers, the exact mechanism remains to be clear, and further studies are warranted to confirm and/or replicate our results.

Our study has several limitations. First, because this is a hospital-based case–control study design, and controls were selected from patients who underwent other surgeries, including those with gastrointestinal conditions, such as colitis, gastric ulcer or polyps, which might also be related to diet and their health, selection bias (i.e., away from the null) has potentially occurred. Next, because dietary information was collected by asking participants what they ate during the past year, recall bias might happen, as the participants might have recalled mistakenly or their diet might have changed during illness. Third, residual confounding might also occur even though we used a comprehensive set of covariates in the multivariable regression models. Fourth, the current analysis focused on lycopene-containing foods, not lycopene per se. Although this molecule is known for its many health-promoting bioactivities, lycopene-containing foods also include other molecules that could contribute to the observed effect. Caution in interpretation should be noted because it is not fully responsible for the protective effect observed in our study. In addition, other nutrients and bioactive compounds found in red/pink foods and vegetables might also be contributing to our findings. We also acknowledge that understanding the impact of a single nutrient or phytochemical, in this case, lycopene, is always challenging when using data from a food frequency questionnaire. Finally, our results might not be easily generalizable to other countries or regions because the current study was conducted in northern Vietnam.

Our study, however, also has several strengths. To our knowledge, again, this is the first study in an Asian, specifically Vietnamese, population that evaluates the relationship between lycopene intake and gastric cancer risk in a sizable sample. Second, the use of FFQ that adopted Vietnamese foods and the Vietnam Food Composition Table provided us reasonably accurate measurement of nutrition intakes, including lycopene intake for the current analysis. Third, the use of a comprehensive set of covariates, on the other hand, helped minimize the potential effects of confounding factors.

## 5. Conclusions

In conclusion, we identified a dose–response inverse association between lycopene intake and risk of gastric cancer in a large case–control study in Vietnam. This inverse association pattern was found regardless of BMI or smoking status, yet observed only among females, ever alcohol drinkers, individuals with *H. pylori* negative status, those without a history of type 2 diabetes and those with blood group B. The results from our study have great implications for a healthy dietary pattern (i.e., lycopene with major sources from fruits and vegetables) for strategies in the prevention and control programs of gastric cancer in low- and middle-income countries.

## Figures and Tables

**Table 1:** Characteristics of All Study Participants by Quintiles of Lycopene Intakes in the Current Case-Control Study.

Characteristics	Quintile 1	Quintile 2	Quintile 3	Quintile 4	Quintile 5	*p*-Value
Lycopene µg/day, mean (SD)	165.1 (98.1)	430.6 (53.8)	611.1 (76.0)	1000.9 (115)	2072.5 (1006)	
Age, mean (SD)	57.7 (11.9)	55.6 (11.6)	55.8 (12.2)	54.4 (12.3)	53.2 (12.3)	<0.001
15–39	64 (7.6)	91 (9.7)	79 (10.9)	104 (12.4)	117 (14.0)	
40–49	140 (16.6)	179 (19.0)	134 (18.5)	180 (21.5)	178 (21.3)	
50–59	250 (29.7)	309 (32.8)	210 (29.0)	254 (30.4)	256 (30.7)	
60–69	254 (30.2)	256 (27.2)	213 (29.5)	209 (25.0)	218 (26.1)	
≥70	134 (15.9)	107 (11.4)	87 (12.0)	89 (10.6)	65 (7.8)	
Sex						
Men	555 (65.9)	591 (62.7)	457 (63.2)	497 (59.4)	480 (57.6)	0.004
Women	287 (34.1)	351 (37.3)	266 (36.8)	339 (40.6)	354 (42.4)	
Highest level of education						
Primary school	179 (21.3)	154 (16.3)	109 (16.1)	122 (14.6)	86 (10.3)	<0.001
Secondary school	406 (48.2)	415 (44.1)	353 (46.8)	345 (41.3)	371 (44.5)	
High school or higher	257 (30.5)	373 (39.6)	261 (36.1)	369 (44.1)	377 (45.2)	
Refrigerator owned						
Yes	554 (64.6)	816 (86.6)	520 (71.9)	672 (80.4)	568 (68.1)	<0.001
No	288 (35.4)	126 (13.4)	203 (28.1)	164 (19.6)	266 (31.9)	
BMI, mean (SD) ^a^	20.1 (3.0)	21.1 (3.0)	20.7 (3.0)	21.0 (3.0)	21 (3.2)	<0.001
<18.5	262	170	177	181	170	
18.5–22.9	410	514	381	438	428	
≥23	128	233	146	207	215	
Family history of cancer						
No	783 (93.0)	863 (91.6)	662 (91.6)	764 (91.4)	758 (90.9)	0.61
Yes	59 (7.0)	79 (8.4)	61 (8.4)	72 (8.6)	76 (9.1)	
Smoking status						
Never smoker	478 (56.8)	519 (55.1)	411 (56.8)	493 (59.0)	522 (62.6)	0.02
Ever smoker	364 (43.2)	423 (44.9)	312 (43.2)	343 (41.0)	312 (37.4)	
Alcohol consumption						
Never drinkers	478 (56.8)	513 (54.5)	389 (53.8)	478 (57.2)	460 (55.2)	0.59
Ever drinkers	364 (43.2)	429 (45.5)	334 (46.2)	358 (42.8)	374 (44.8)	
Coffee drinking status						
No	707 (84.0)	743 (78.9)	537 (74.3)	611 (73.1)	613 (73.5)	<0.001
Yes	135 (16.0)	199 (21.1)	186 (25.7)	225 (26.9)	221 (26.5)	
History of type 2 diabetes						
No	721 (96.6)	817 (93.7)	600 (95.5)	687 (92.7)	632 (94.3)	0.008
Yes	25 (3.4)	55 (6.3)	28 (4.5)	54 (7.3)	38 (5.7)	
Total energy intake (Kcal/day), mean (SD)	1563.5 (435.4)	1589.2 (426)	1698.3 (412.1)	1735.3 (412.6)	1872.2 (468.3)	<0.001
Blood group ^a^						
A	152 (23.5)	167 (20.8)	139 (24.7)	155 (22.9)	122 (21.0)	0.78
AB	27 (4.2)	47 (5.9)	25 (4.4)	36 (5.3)	31 (5.3)	
B	181 (28.0)	235 (29.3)	156 (27.8)	207 (30.6)	170 (29.3)	
O	287 (44.4)	352 (43.9)	242 (43.1)	279 (41.2)	257 (44.3)	
*H. pylori* infection ^a^						
Negative	202 (44.1)	173 (41.9)	140 (37.0)	175 (39.0)	137 (37.5)	0.17
Positive	256 (55.9)	240 (58.1)	238 (63.0)	274 (61.0)	228 (62.5)	

^a^ Based on reported data. Abbreviations: BMI; body mass index; SD; standard deviation.

**Table 2:** Characteristics of Study Participants in the Current Case-Control Study [31].

Characteristics	Total (N = 4177)	Cancer (n = 1182)	Controls (n = 2995)	*p*-Value
Lycopene intake µg/day, mean (SD)	4.9 (2.3)	4.3 (2.1)	5.2 (2.3)	<0.001
Age, mean (SD)	55.36 (12.13)	57.6 (11.5)	54.5 (12.2)	<0.001
15–39	455	88 (7.4)	367 (12.3)	
40–49	811	189 (10.0)	622 (20.8)	
50–59	1279	363 (30.7)	916 (30.6)	
60–69	1150	369 (31.2)	781 (26.1)	
≥70	482	173 (14.6)	309 (10.3)	
Sex				
Men	2580	823 (69.6)	1757 (58.7)	<0.001
Women	1597	359 (30.4)	1238 (41.3)	
Highest level of education				
Primary school	650	208 (17.6)	442 (14.8)	<0.001
Secondary school	1890	568 (48.1)	1322 (44.1)	
High school or higher	1637	406 (34.3)	1231 (41.1)	
Refrigerator owned				
Yes	3130	764 (64.6)	2366 (79.0)	<0.001
No	1047	418 (35.4)	629 (21.0)	
BMI, mean (SD) ^a^	20.79 (3.05)	19.4 (2.8)	21.3 (3)	
<18.5	960	469 (41.5)	491 (16.8)	<0.001
18.5–22.9	2171	541 (47.8)	1630 (55.7)	
≥23	929	121 (10.7)	808 (27.6)	
Family history of cancer				
No	3830	1066 (90.2)	2764 (92.3)	0.03
Yes	347	116 (9.8)	231 (7.7)	
Smoking status				
Never smoker	2423	601 (50.8)	1822 (60.8)	<0.001
Ever smoker	1754	581 (49.2)	1173 (39.2)	
Alcohol consumption				
Never drinkers	2318	612 (51.8)	1706 (57.0)	<0.001
Ever drinkers	1859	570 (48.2)	1289 (43.0)	
Coffee drinking status				
Never drinker	3211	922 (78.0)	2289 (76.4)	<0.001
Ever drinker	966	260 (22.0)	706 (23.6)	
History of type 2 diabetes				
Yes	200	29 (2.9)	171 (6.5)	<0.001
No	3457	878 (97.1)	2479 (93.5)	
Histologic type				
Non-cardia	1145	1145 (96.9)	-	
Cardia	37	37 (3.1)	-	
Total energy intake (Kcal/day), mean (SD)	1688.63 (445.86)	1650.6 (455)	1703.6 (441.4)	
Tertile 1	1396	423 (35.8)	973 (32.5)	<0.001
Tertile 2	1390	393 (33.2)	1032 (34.5)	
Tertile 3	1391	366 (31.0)	990 (33.1)	
Blood group ^a^				
A	735	258 (26.6)	477 (20.8)	0.001
AB	166	55 (5.7)	111 (4.8)	
B	949	278 (28.5)	671 (29.2)	
O	1417	380 (39.1)	1037 (45.2)	
*H. pylori* infection ^a^				
Negative	827	265 (39.8)	562 (40.2)	0.88
Positive	1236	400 (60.2)	836 (59.8)	

^a^ Based on available data, SD is standard deviation, and BMI is body mass index (Asian category, kg/m^2^).

**Table 3:** Association Between Lycopene Intake and Gastric Cancer Risk, Overall and Stratified by Sex in the Current Case-Control Study.

Lycopene Intake (Mean: µg/Day)	Case	Control	OR (95% CI) *	OR (95% CI) *
Overall				
Quintile 1 (165.1)	331	511	1.00	1.00
Quintile 2 (430.6)	231	711	0.50 (0.41–0.61)	0.63 (0.51–0.79)
Quintile 3 (611.1)	202	521	0.60 (0.48–0.74)	0.64 (0.51–0.80)
Quintile 4 (1000.9)	211	625	0.52 (0.42–0.64)	0.65 (0.52–0.81)
Quintile 5 (2072.5)	207	627	0.51 (0.41–0.63)	0.62 (0.50–0.78)
Continuous (per SD increment)	1182	2995	0.83 (0.77–0.90)	0.88 (0.81–0.95)
*P_trend_*			<0.001	0.002
Men				
Quintile 1 (163.7)	234	321	1.00	1.00
Quintile 2 (429.8)	162	429	0.52 (0.40–0.66)	0.63 (0.48–0.82)
Quintile 3 (613.1)	144	313	0.63 (0.49–0.82)	0.66 (0.50–0.87)
Quintile 4 (992.6)	137	360	0.52 (0.40–0.68)	0.64 (0.48–0.84)
Quintile 5 (2062.1)	146	334	0.60 (0.46–0.78)	0.73 (0.55–0.96)
Continuous (per SD increment)	823	1757	0.88 (0.80–0.96)	0.93 (0.84–1.02)
*P_trend_*			0.006	0.14
Women				
Quintile 1 (167.8)	97	190	1.00	1.00
Quintile 2 (432.0)	69	282	0.48 (0.33–0.69)	0.62 (0.42–0.91)
Quintile 3 (607.8)	58	208	0.55 (0.37–0.80)	0.58 (0.39–0.87)
Quintile 4 (1013.1)	74	265	0.55 (0.38–0.78)	0.67 (0.46–0.98)
Quintile 5 (2086.6)	61	293	0.41 (0.28–0.59)	0.47 (0.32–0.70)
Continuous (per SD increment)	359	1238	0.77 (0.67–0.89)	0.80 (0.69–0.92)
*P_trend_*			<0.001	<0.001
*P_heterogeneity_*			0.13	0.13

* Model adjusted for age groups (15–39, 40–49, 50–59, 60–69 and ≥70), sex (if applicable), the highest education level (primary, secondary, high school or higher), BMI (kg/m^2^, <18.5, 18.5- < 23, ≥23), alcohol consumption (yes/no), family history of cancer (yes/no), smoking status (ever/never), history of diabetes (yes/no), coffee drinking (yes/no), total energy intake (kcal/day, tertile), fridge at home, blood group (A, AB, B, O), four periods of data collection, and *H. pylori* status. Abbreviations: CI: confidence interval; OR: odds ratio; SD: Standard deviation.

**Table 4:** Association Between Lycopene Intake and Gastric Cancer Risk, Stratified by BMI, Smoking Status, Alcohol Drinking Status, History of Diabetes, *H. pylori* Infection Status and Blood Group in the Current Case-Control Study.

Lycopene Intake (Mean: µg/Day)	Case	Control	OR (95% CI) *	OR (95% CI) *
Cardia				
Quartile 1 (165.1)	12	511	1.00	1.00
Quartile 2 (509.0)	13	1232	0.45 (0.20–0.99)	0.62 (0.27–1.42)
Quartile 3 (1000.9)	7	625	0.48 (0.19–1.22)	0.62 (0.23–1.65)
Quartile 4 (2072.5)	5	627	0.34 (0.12–0.97)	0.45 (0.15–1.35)
Continuous (per SD increment)	37	2995	0.55 (0.32–0.96)	0.61 (0.36–1.04)
*P_trend_*			0.04	0.06
Non-cardia				
Quintile 1 (164.9)	319	511	1.00	1.00
Quintile 2 (430.5)	224	711	0.50 (0.41–0.62)	0.65 (0.52–0.81)
Quintile 3 (611.3)	196	521	0.60 (0.49–0.75)	0.65 (0.51–0.81)
Quintile 4 (1001.3)	204	625	0.52 (0.42–0.65)	0.66 (0.53–0.83)
Quintile 5 (2076.7)	202	627	0.52 (0.42–0.64)	0.63 (0.50–0.80)
Continuous (per SD increment)	1145	2995	0.84 (0.78–0.91)	0.89 (0.82–0.96)
*P_trend_*			<0.001	0.004
BMI < 23 kg/m^2^				
Quintile 1 (164.3)	276	396	1.00	1.00
Quintile 2 (429.2)	199	485	0.59 (0.47–0.74)	0.71 (0.56–0.90)
Quintile 3 (610.2)	179	379	0.68 (0.54–0.86)	0.72 (0.57–0.93)
Quintile 4 (998.2)	182	437	0.60 (0.47–0.75)	0.73 (0.57–0.94)
Quintile 5 (2056.1)	174	424	0.59 (0.47–0.74)	0.69 (0.53–0.88)
Continuous (per SD increment)	1010	2121	0.87 (0.80–0.94)	0.91 (0.83–0.99)
*P_trend_*			0.001	0.03
BMI ≥ 23 kg/m^2^				
Quintile 1 (168.2)	55	115	1.00	1.00
Quintile 2 (434.4)	32	226	0.30 (0.18–0.48)	0.36 (0.21–0.61)
Quintile 3 (614.3)	23	142	0.34 (0.20–0.58)	0.36 (0.20–0.64)
Quintile 4 (1008.9)	29	188	0.32 (0.19–0.54)	0.39 (0.23–0.67)
Quintile 5 (2114.2)	33	203	0.34 (0.21–0.55)	0.42 (0.25–0.73)
Continuous (per SD increment)	172	874	0.73 (0.59–0.89)	0.78 (0.63–0.96)
*P_trend_*			0.003	0.02
*P_heterogeneity_*			0.58	0.45
Never smoker				
Quintile 1 (167.3)	177	301	1.00	
Quintile 2 (429.1)	110	409	0.46 (0.35–0.61)	0.61 (0.45–0.82)
Quintile 3 (611.1)	100	311	0.55 (0.41–0.73)	0.62 (0.45–0.84)
Quintile 4 (1006.6)	102	391	0.44 (0.33–0.59)	0.57 (0.42–0.77)
Quintile 5 (2140.5)	112	410	0.46 (0.35–0.61)	0.59 (0.43–0.80)
Continuous (per SD increment)	601	1822	0.81 (0.73–0.90)	0.85 (0.77–0.95)
*P_trend_*			<0.001	0.004
Ever smoker				
Quintile 1 (162.2)	154	210	1.00	1.00
Quintile 2 (432.6)	121	302	0.55 (0.41–0.73)	0.66 (0.49–0.91)
Quintile 3 (611.1)	102	210	0.66 (0.48–0.91)	0.68 (0.49–0.94)
Quintile 4 (992.8)	109	234	0.64 (0.47–0.86)	0.78 (0.56–1.08)
Quintile 5 (2019.0)	95	217	0.60 (0.43–0.82)	0.66 (0.47–0.92)
Continuous (per SD increment)	581	1173	0.88 (0.78–0.99)	0.91 (0.80–1.02)
*P_trend_*			0.03	0.11
*P_heterogeneity_*			0.32	0.42
Never drinker				
Quintile 1 (168.1)	174	304	1.00	1.00
Quintile 2 (427.2)	113	400	0.49 (0.37–0.65)	0.66 (0.49–0.89)
Quintile 3 (610.9)	98	291	0.59 (0.44–0.79)	0.61 (0.45–0.83)
Quintile 4 (1004.6)	115	363	0.55 (0.42–0.73)	0.67 (0.50–0.91)
Quintile 5 (2066.1)	112	348	0.56 (0.42–0.75)	0.66 (0.48–0.89)
Continuous (per SD increment)	612	1706	0.89 (0.81–0.99)	0.92 (0.83–1.02)
*P_trend_*			0.03	0.12
Ever drinker				
Quintile 1 (161.1)	157	207	1.00	1.00
Quintile 2 (434.8)	118	311	0.50 (0.37–0.67)	0.58 (0.42–0.79)
Quintile 3 (611.4)	104	230	0.60 (0.44–0.81)	0.62 (0.44–0.86)
Quintile 4 (996.1)	96	262	0.48 (0.35–0.66)	0.59 (0.42–0.83)
Quintile 5 (2080.4)	95	279	0.45 (0.33–0.61)	0.57 (0.41–0.80)
Continuous (per SD increment)	570	1289	0.76 (0.67–0.86)	0.83 (0.73–0.94)
*P_trend_*			<0.001	0.003
*P_heterogeneity_*			0.05	0.05
*H. pylori* negative				
Quintile 1 (165.0)	84	118	1.00	1.00
Quintile 2 (434.1)	51	122	0.59 (0.38–0.90)	0.98 (0.58–1.66)
Quintile 3 (610.8)	47	93	0.71 (0.45–1.11)	0.74 (0.43–1.28)
Quintile 4 (1012.1)	43	132	0.46 (0.29–0.71)	0.56 (0.33–0.98)
Quintile 5 (2169.0)	40	97	0.58 (0.36–0.92)	0.65 (0.37–1.15)
Continuous (per SD increment)	265	562	0.80 (0.67–0.95)	0.77 (0.62–0.95)
*P_trend_*			0.01	0.02
*H. pylori* positive				
Quintile 1 (163.5)	108	148	1.00	1.00
Quintile 2 (427.3)	78	162	0.66 (0.46–0.95)	1.29 (0.82–2.03)
Quintile 3 (615.3)	80	158	0.69 (0.48–1.00)	0.89 (0.57–1.38)
Quintile 4 (1005.7)	71	203	0.48 (0.33–0.69)	0.85 (0.54–1.34)
Quintile 5 (2019.6)	63	165	0.52 (0.36–0.77)	0.65 (0.40–1.05)
Continuous (per SD increment)	400	836	0.80 (0.69–0.93)	0.86 (0.72–1.02)
*P_trend_*			0.003	0.08
*P_heterogeneity_*			0.26	0.98
No diabetes				
Quintile 1 (168.0)	283	438	1.00	1.00
Quintile 2 (434.0)	194	623	0.48 (0.39–0.60)	0.61 (0.49–0.78)
Quintile 3 (607.6)	166	434	0.59 (0.47–0.75)	0.64 (0.50–0.82)
Quintile 4 (1002.5)	170	517	0.51 (0.40–0.64)	0.66 (0.52–0.84)
Quintile 5 (2070.4)	165	467	0.55 (0.43–0.69)	0.64 (0.50–0.82)
Continuous (per SD increment)	978	2479	0.85 (0.78–0.93)	0.88 (0.81–0.97)
*P_trend_*			<0.001	0.006
Blood Group				
Blood Group A				
Quintile 1 (162.8)	80	72	1.00	1.00
Quintile 2 (434.5)	45	122	0.33 (0.21–0.53)	0.37 (0.22–0.61)
Quintile 3 (606.9)	45	94	0.43 (0.27–0.69)	0.35 (0.21–0.60)
Quintile 4 (1003.8)	41	114	0.32 (0.20–0.52)	0.38 (0.22–0.63)
Quintile 5 (2168.4)	47	75	0.56 (0.35–0.92)	0.64 (0.37–1.10)
Continuous (per SD increment)	258	477	0.54 (0.42–0.70)	0.93 (0.77–1.12)
*P_trend_*			<0.001	0.45
Blood Group AB				
Quintile 1 (178.0)	8	19	1.00	1.00
Quintile 2 (432.7)	17	30	1.35 (0.49–3.72)	1.99 (0.56–7.05)
Quintile 3 (650.3)	10	15	1.58 (0.50–5.00)	1.84 (0.44–7.61)
Quintile 4 (1021.7)	7	29	0.57 (0.18–1.84)	1.24 (0.30–5.15)
Quintile 5 (2046.8)	13	18	1.72 (0.58–5.11)	2.61 (0.65–10.42)
Continuous (per SD increment)	55	111	1.05 (0.75–1.46)	1.24 (0.82–1.87)
*P_trend_*			0.78	0.31
Blood Group B				
Quintile 1 (169.9)	77	104	1.00	1.00
Quintile 2 (430.8)	60	175	0.46 (0.31–0.70)	0.68 (0.42–1.08)
Quintile 3 (610.6)	50	106	0.64 (0.41–1.00)	0.82 (0.50–1.35)
Quintile 4 (1004.3)	50	157	0.43 (0.28–0.66)	0.68 (0.42–1.11)
Quintile 5 (2089.6)	41	129	0.43 (0.27–0.68)	0.51 (0.30–0.87)
Continuous (per SD increment)	278	671	0.77 (0.65–0.92)	0.81 (0.68–0.97)
*P_trend_*			0.004	0.02
Blood Group O				
Quintile 1 (174.2)	102	185	1.00	1.00
Quintile 2 (435.0)	81	271	0.54 (0.38–0.77)	0.90 (0.61–1.34)
Quintile 3 (602.7)	60	182	0.60 (0.41–0.87)	0.74 (0.48–1.14)
Quintile 4 (1008.3)	74	205	0.65 (0.46–0.94)	0.92 (0.61–1.38)
Quintile 5 (2028.5)	63	194	0.59 (0.41–0.86)	0.72 (0.47–1.10)
Continuous (per SD increment)	380	1037	0.88 (0.76–1.01)	0.90 (0.78–1.05)
*P_trend_*			0.06	0.17
*P_heterogeneity_*			0.29	0.22

* Model adjusted for age groups (15–39, 40–49, 50–59, 60–69 and ≥70), sex, the highest education level (primary, secondary, high school or higher), BMI (kg/m^2^, <18.5, 18.5- < 23, ≥23), alcohol consumption (yes/no) (if applicable), family history of cancer (yes/no), smoking status (ever/never) (if applicable), history of diabetes (yes/no), coffee drinking (yes/no), total energy intake (kcal/day, tertile), fridge at home, blood group (A, AB, B, O), four periods of data collection, and *H. pylori* status (if applicable). Abbreviations: CI: confidence interval; OR: odds ratio; SD: standard deviation.

## Data Availability

Data is available from the corresponding authors upon reasonable request.

## Supplementary Materials

The following supporting information can be downloaded at: https://www.mdpi.com/article/10.3390/nu18071143/s1, Table S1. Association Between Lycopene Intake and Overall Gastric Cancer, Models with Additionally Adjusted for Fruits, Vegetables, and Both.

## Abbreviations

The following abbreviations are used in this manuscript:

BMI	Body mass index
CHS	Commune health station
CI	Confidence interval
DLQ	Demographic lifestyle questionnaire
FFQ	Food frequency questionnaire
ICD-10	International Classification of Disease, Ten Revision
HR	Hazard ratio
LMICs	Low- and middle-income countries
OR	Odds ratio
WHO	World Health Organization
