# Supplementary material for: Dietary Lycopene Intake and Gastric Cancer Risk: Findings from a Case-Control Study
Source: Nutrients. 2026 Apr 2;18(7):1143. doi: 10.3390/nu18071143 (PMC13074409; doi:10.3390/nu18071143)
Supplement: Supplementary file 1 [file nutrients-18-01143-s001.zip › nutrients-4183256-supplementary.pdf]

Supplementary Table S1. Association Between Lycopene Intake and Overall Gastric Cancer, Models with Additionally Adjusted for Fruits, Vegetables, and Both

Lycopene Intake (Mean: µg/day)	Case	Control	OR (95% CI)#	OR (95% CI)$	OR (95% CI)*
Overall					
Quintile 1 (165.1)	331	511	1.00	1.00	
Quintile 2 (430.6)	231	711	0.44 (0.33, 0.59)	0.42 (0.32, 0.55)	0.41 (0.31, 0.55)
Quintile 3 (611.1)	202	521	0.44 (0.31, 0.62)	0.42 (0.30, 0.59)	0.45 (0.31, 0.65)
Quintile 4 (1000.9)	211	625	0.45 (0.32, 0.63)	0.43 (0.31, 0.59)	0.47 (0.33, 0.66)
Quintile 5 (2072.5)	207	627	0.44 (0.28, 0.68)	0.42 (0.29, 0.60)	0.50 (0.32, 0.80)
Continuous (per SD increment)	1,182	2,995	0.82 (0.69, 0.98)	0.80 (0.70, 0.91)	0.81 (0.66, 0.99)
P_{trend}			<0.001	<0.001	0.002

Model adjusted for age groups (15-49, 50-59, 60+), sex (if applicable), the highest education level (primary, secondary, high school or higher), BMI (kg/m^2, <18.5, 18.5-<23, ≥23), alcohol consumption (yes/no), family history of cancer (yes/no), smoking status (ever/never), history of diabetes (yes/no), coffee drinking (yes/no), total energy intake (kcal/day, tertile), fridge at home, blood group (A, AB, B, O), four periods of data collection, *H. Pylori* status, and fruits consumption (quintile);

$ Model adjusted (#) and plus vegetables (quintile)

* Model adjusted (#) and plus vegetables (quintile) and fruits consumption (quintile);

Abbreviations: CI: confidence interval; OR: odds ratio; SD: Standard deviation.
